# Big data need big theory too

**DOI:** 10.1098/rsta.2016.0153

**Published:** 2016-11-13

**Authors:** Peter V. Coveney, Edward R. Dougherty, Roger R. Highfield

**Affiliations:** 1 Centre for Computational Science, University College London, Gordon Street, London WC1H 0AJ, UK; 2 Center for Bioinformatics and Genomic Systems Engineering, Texas A&M University, College Station, TX 77843-31283, USA; 3 Science Museum, Exhibition Road, London SW7 2DD, UK

**Keywords:** machine learning, big data, personalized medicine, biomedicine, epistemology

## Abstract

The current interest in big data, machine learning and data analytics has generated the widespread impression that such methods are capable of solving most problems without the need for conventional scientific methods of inquiry. Interest in these methods is intensifying, accelerated by the ease with which digitized data can be acquired in virtually all fields of endeavour, from science, healthcare and cybersecurity to economics, social sciences and the humanities. In multiscale modelling, machine learning appears to provide a shortcut to reveal correlations of arbitrary complexity between processes at the atomic, molecular, meso- and macroscales. Here, we point out the weaknesses of pure big data approaches with particular focus on biology and medicine, which fail to provide conceptual accounts for the processes to which they are applied. No matter their ‘depth’ and the sophistication of data-driven methods, such as artificial neural nets, in the end they merely fit curves to existing data. Not only do these methods invariably require far larger quantities of data than anticipated by big data aficionados in order to produce statistically reliable results, but they can also fail in circumstances beyond the range of the data used to train them because they are not designed to model the structural characteristics of the underlying system. We argue that it is vital to use theory as a guide to experimental design for maximal efficiency of data collection and to produce reliable predictive models and conceptual knowledge. Rather than continuing to fund, pursue and promote ‘blind’ big data projects with massive budgets, we call for more funding to be allocated to the elucidation of the multiscale and stochastic processes controlling the behaviour of complex systems, including those of life, medicine and healthcare.

This article is part of the themed issue ‘Multiscale modelling at the physics–chemistry–biology interface’.

## 1. Introduction

Whether from telescopes gazing at the night sky, the detectors of the Large Hadron Collider (LHC), cameras trained on professional footballers or sequencers generating genomes, it has become increasingly easy to acquire large datasets. Does this mean, as popular commentators have speculated, the end of theory and the scientific method [1]? Unlikely. Do more data at least mean that we can more easily fathom Nature's mysteries? Not necessarily.

The issue is not data *per se*, but the manner in which data are collected, curated and integrated into scientific modelling. Modern science is grounded in an epistemology whose essentials were laid down in the first half of the seventeenth century with Sir Francis Bacon's insistence on experimental design and Galileo's establishing scientific knowledge on a mathematical basis.

Modern science is neither strict empiricism nor strict rationalism; rather, it is both, an integration of empiricism and rationalism with respect to experimental design, predictive modelling and verification. In the words of Immanuel Kant: ‘Perception without conception is blind; conception without perception is empty’ [2].

The rise of big data in medicine not only has huge implications for public health [3] but is also remarkable, because it heralds a profound change in the way that science is done. In this paper, we shall examine the ability of research in the biological and medical sciences to provide valid scientific knowledge and to make predictions, not only those that are novel but those that are actionable. Some are actionable in a broad sense, such as helping to hone an industrial process over many iterations. A more demanding sense in which predictions are actionable arises when there is a window of opportunity to predict the future, so action can be taken before it becomes a reality, as is already the case when forecasting severe weather. In medicine, the most vivid example of an actionable prediction is one that can change the future condition of a patient, whether to pick one antimicrobial drug in preference to another when confronted with a severe infection or to select the best approach for risky life-saving surgery. We provide evidence that the successful use of big data in these demanding applications depends on more than the quantity of data alone and are sceptical that a purely data-driven approach—‘blind big data’—can deliver the high expectations of some of its most passionate proponents.

## 2. Modern science

Modern science was born in the seventeenth century as a fusion of observation and reason. Radical empiricism (data without reason) and rationalism (reason without data) were rejected in the quest for knowledge of Nature.

The matter is articulated by Sir Francis Bacon in his *Novum Organum* (1620)

Those who have handled sciences have been either men of experiment or men of dogmas. The men of experiment are like the ant, they only collect and use; the reasoners resemble spiders, who make cobwebs out of their own substance. But the bee takes a middle course: it gathers its material from the flowers of the garden and of the field, but transforms and digests it by a power of its own. [4]

The new science will be that of the bee: the senses will gather up data from the world and the mind will transform and organize. With the general schema given by the metaphor of the bees, Bacon addresses how the data are to be obtained

There remains simple experience which, if taken as it comes, is called accident; if sought for, experiment. But this kind of experience is no better than a broom without its band, as the saying is—a mere groping, as of men in the dark, that feel all round them for the chance of finding their way, when they had much better wait for daylight, or light a candle, and then go. But the true method of experience, on the contrary, first lights the candle, and then by means of the candle shows the way; commencing as it does with experience duly ordered and digested, not bungling or erratic, and from it educing axioms, and from established axioms again new experiments. [4]

Data are not to be obtained haphazardly, ‘groping, as of men in the dark’, but guided by reason. Here is the basic scientific method: reason guiding experiment, new reason and then experiment.

As to the proper form and content of scientific theories, this would be clarified by Galileo in his *Dialogues Concerning Two New Sciences* (1632)

The present does not seem to me to be an opportune time to enter into the investigation of the cause of the acceleration of natural motion, concerning which various philosophers have produced various opinions…. Such fantasies, and others like them, would have to be examined and resolved, with little gain. For the present, it suffices our Author that we understand him to want us to investigate and demonstrate some attributes of a motion so accelerated. [5]

Science concerns the mathematical description of behaviour. We will not waste time constructing ‘fantasies’ with which to explain acceleration in terms of causality. There may or may not be such a thing as causality, but that is irrelevant. Causality is not part of science, whose business is to ‘investigate and demonstrate some attributes of a motion’.

What is behind these mathematical formulae? We do not know and it does not matter. With regard to gravity, in *Dialogue Concerning the Two Chief World Systems* (1632), Galileo writes, ‘You are wrong, Simplicio; you should say that everyone knows that it is called “gravity”. But I am not asking you for the name, but the essence of the thing…. We don't really understand what principle or what power it is that moves a stone downwards' [6]. We observe that bodies fall and their properties can be characterized mathematically. Beyond that, it is just words.

The basic composition of a scientific theory is summarized in *Philosophiæ Naturalis Principia Mathematica* (1687), where Sir Isaac Newton writes

For I here design only to give a mathematical notion of these forces, without considering their physical causes and seats…. Hitherto I have not been able to discover the cause of those properties of gravity from the phenomena, and I frame no hypothesis; for whatever is not deduced from the phenomena is to be called an hypothesis; and hypotheses, whether metaphysical or physical, whether of occult qualities or mechanical, have no place in experimental philosophy. [7]

‘Hypotheses non fingo’—‘I frame no hypotheses’. This is the fundamental principle of modern science. Does gravity exist? Something exists, but its substance is beyond our ken. Is Nature the product of cause and effect? We do not know and science has no interest in the question.

The full implications of the seventeenth century revolution in scientific epistemology did not become apparent until the twentieth century, with the coming of relativity theory and quantum mechanics. In particular, validation of a scientific theory seemed straightforward: just run some tests and see if the predictions of the theory hold up. This becomes difficult when the mathematical theory is divorced from ordinary human physical understanding. The extent of this divorce is apparent in the words of Erwin Schrödinger when he considers modelling the quantum theory based on ordinary human experience. He writes

A completely satisfactory model of this type is not only practically inaccessible, but not even thinkable. Or, to be precise, we can, of course, think it, but however we think it, it is wrong; not perhaps quite as meaningless as a ‘triangular circle’, but much more so than a ‘winged lion’. [8]

The startling fact is that our ability to model Nature far outstrips our ability to conceive it; indeed, only the most mundane aspects of Nature are intelligible, those corresponding to our everyday experience. Formation of a scientific theory requires a mathematical conceptualization characterizing the observations. Validation requires aligning the terms of the theory with phenomena, making predictions from the theory, designing confirmatory experiments, and then checking to see if the predictions are sufficiently consistent with the experimental observations.

To summarize, an acceptable modern scientific theory must satisfy four properties:

1. It takes the form of a model formulated in a mathematical system.
2. Precise relationships are specified between terms in the theory and measurements of corresponding events.
3. There are validating experimental data—that is, a set of future quantitative predictions derived from the theory and the corresponding measurements.
4. A rigorous statistical analysis supports acceptance of the theory based on concordance between the predictions and the measurements.

The last property is extremely important in theories concerning complex stochastic systems, such as are required in biology.

In addition, if it is to be useful, an acceptable modern theory must be able to turn data into predictions at a later time before those predictions become a reality. If, for example, data about a patient are to be used to guide medical care, then predictions about the impact of various treatments have to be both accurate and timely.

However, the biological and medical sciences do not neatly fit into this simple paradigm of today's science. These fields are rational but in the sense that, like pre-Galilean science, they typically rationalize observations after the event. In the era of big data, with the rise of blind data gathering, the biological and medical sciences increasingly recapitulate pre-Baconianism by placing undue emphasis on blind data gathering without even *post hoc* explanations.

This rise of a modern version of pre-seventeenth century thinking, which places too much emphasis on the power of haphazardly gathered data, is problematic for the following reasons.

First, not all data are reliable. The fact that ‘most published research findings are false’, as famously reported by John Ioannidis in *PLOS Medicine* [9], suggests one important dataset—the conclusions of peer-reviewed studies—consists of predominantly bad data and cannot be relied upon without evidence of good experimental design and rigorous statistical analysis.

Second, we need models and theoretical insights to help guide the collection, curation and interpretation of data. The relatively meagre initial returns from the human genome demonstrate that data do not translate readily into understanding, let alone treatments. Even using a rigorous predictive statistical framework, characterizing average behaviour from big genomics data will not deliver ‘personalized medicine’. This is now acknowledged by the rise of ‘precision medicine’, which aims to link genes with pathologies in stratified populations.

Third, we have to take care when extrapolating beyond the range of existing data. The overestimate of peak influenza levels by Google flu trends [10] reminds us that past success in describing epidemics is no guarantee of future performance.

Fourth, correlations observed in different sets of data are not necessarily evidence of dependency. The problem of spurious correlations is familiar when it comes to the use of quantitative structure–activity relationship models and machine learning to predict the biological activity of molecules [11].

**Figure 1.**
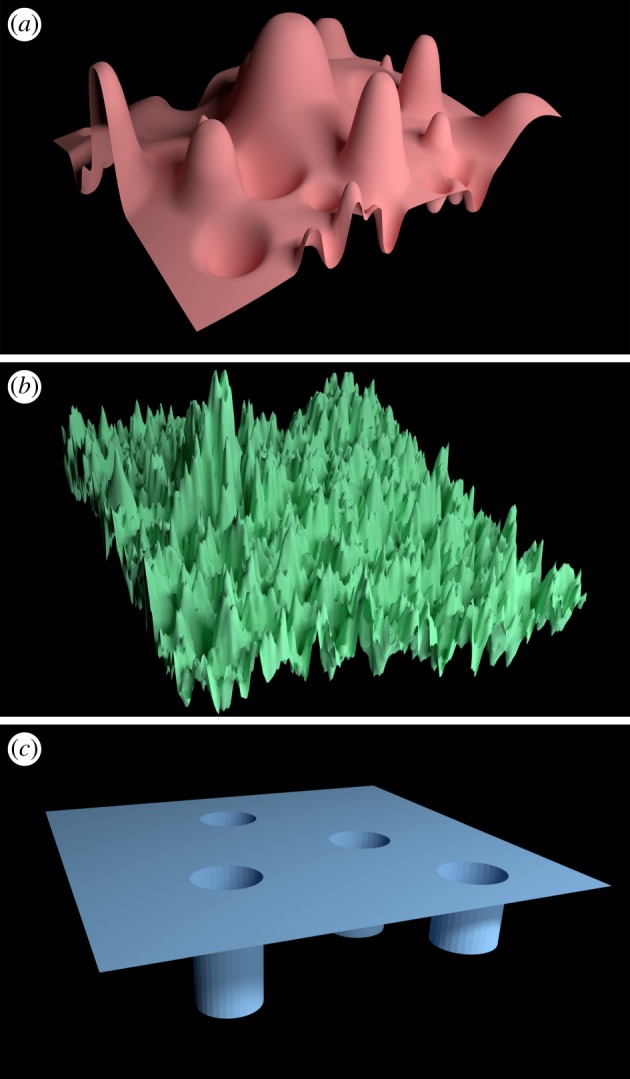
Three-dimensional plots that illustrate the way in which properties of a system (the ‘landscape’), here shown for two variables in order to be able to visualize it, can defeat machine learning approaches in some circumstances. (*a*) For simple situations where the landscape is relatively smooth and the number of such variables is small (two are shown here for simplicity, though the number is usually far greater), machine learning methods can be expected to do a good job of predicting behaviour over the domain in which they have been trained, just as it is easy to trace a smoothly changing slope. (*b*) When the landscape has more complex and rapidly varying (including e.g. fractal) character, or (*c*) has a ‘pathological’ form (essentially featureless except for a few controlling singularities), such learning activities are prone to fail without a level of data coverage which is too dense to be practically feasible. Extrapolation is unreliable in all instances; interpolation is also hazardous in (*b*,*c*).

Fifth, we must respect the sensitivity of complex systems to tiny errors in data and the effects of chaos. Some of the first insights into chaos came from a biological discipline—ecology [12,13]—yet these insights are sometimes neglected. Performing future drug discovery through machine learning based on accessing all known drugs and pre-existing molecular targets is liable to fail because it is based on historical chemical structures while tiny structural changes in ligands can lead to dramatic differences in potency (cf. figure 1, where the situation is most akin to (*b*) and does not include scope for reliable predictive capability in the case of new protein targets and compounds).

Sixth, if this endeavour is to be useful, we need to be able to turn data into true predictions, that is, predictions of events in novel circumstances, or predictions of events before they occur (not *post hoc* explanations).

## 3. Radical empiricism (big data)

In this, the age of data, researchers are using machine learning to make sense of the rising tide of bits and bytes. This approach offers a way to correlate events at different length and time scales, leapfrogging the problems of applying multiscale modelling and simulation in a Popperian manner. However, no matter their ‘depth’ and sophistication, machine learning algorithms merely fit model forms to data. They may be capable of effective interpolation, but not of extrapolation beyond their training domain. They offer no structural explanations of the correlations they reveal, many of which are likely to be false-positives [14]. They remind us of Hans Reichenbach's comment, ‘A radical empiricism … denies the possibility of knowledge’ [15].

One can visualize machine learning's hunt for solutions on a landscape—the cost function—drawn over a high-dimensional feature space in which all variables are plotted on orthogonal axes; the tallest peak or deepest valley of the cost function defines the optimal solution. The larger the space, the more data required. Popular methods such as distribution-free inference are usually ineffective unless the problem is simple and the sample size huge. This problem has been extensively studied in pattern recognition, where complex models commonly overfit the data, meaning that, while accounting for the existing data, they do not perform well on new data [16]. The problem is aggravated for small samples, because accurate distribution-free error estimation is usually impossible and accuracy can only be assessed with distributional assumptions, which are not present [17].

In the simplified examples shown in figure 1, one can intuitively understand how a random sample of measurements of height above sea level across a golf course is unlikely to reveal the all-important locations of the holes, and how curve-fitting methods will struggle to map the recursive nature of a fractal surface.

Deep theory requires deep thinking, for both discovery and validation. Writing with Leopold Infeld, Albert Einstein explained how thinking had evolved in their book *The Evolution of Physics: The Growth of Ideas From Early Concepts to Relativity and Quanta*:

In nearly every detective novel since the admirable stories of Conan Doyle there comes a time when the investigator has collected all the facts he needs for at least some phase of his problem. These facts often seem quite strange, incoherent, and wholly unrelated. The great detective, however, realizes that no further investigation is needed at the moment, and that only pure thinking will lead to a correlation of the facts collected. So he plays his violin, or lounges in his armchair enjoying a pipe, when suddenly, by Jove, he has it! Not only does he have an explanation for the clues at hand but he knows that certain other events must have happened. Since he now knows exactly where to look for it, he may go out, if he likes, to collect further confirmation for his theory. [18]

Given Bacon's analogy, one is reminded of the story of the blind men and the elephant: local data are difficult to interpret without a mental model of a pachyderm. In a similar way, various big data initiatives are blindly groping about that great beast that we know as biology. We need theory to help envisage it in all its glory.

In a system with significant complexity, it becomes daunting to get enough data for meaningful conclusions. Two decades ago, one author (P.V.C.) used what are now called ‘big data’ methods to predict thickening times of complex slurries from infrared spectra of cement powders. Even though this became a commercial offering [19], it has not brought us one iota closer to understanding what mechanisms are at play, and thus it has not provided us with the insights necessary to develop new kinds of materials with novel properties. As for the dream of minting new physical laws via data mining, the results to date are limited [20,21] and unconvincing [22]. Regarding deep laws, one of us (E.R.D.) has previously asked: ‘Does anyone really believe that data mining could produce the general theory of relativity?’ [23].

The most profound challenge arises in medicine and biology, because big data are actually tiny relative to the complexity of systems in these fields. This ‘curse of dimensionality’ subsumes what in statistics are called the peaking and multiple comparisons problems. Fortunately, it can be broken. Although the discovery of the Higgs boson at CERN's LHC required petabytes of data, physicists used theory to initiate and guide their search for the missing mass-generating particle within the Standard Model. Higgs and Englert shared the 2013 Nobel Prize in Physics, because their theories, demonstrating the necessary existence of this particle, underpinned the multibillion dollar enterprise. In the same way, we do not predict tomorrow's weather by averaging historic records of that day's weather. Mathematical models (essentially solving Navier–Stokes equations) do a much better job with real-time data from satellites and other sensors.

Many advocates of big data in biology still hope that we will not need theory to understand the basis of health and disease. However, even using a rigorous predictive statistical framework, characterizing average behaviour from big data will not deliver ‘personalized medicine’. Trying to forecast a patient's reaction to a drug based on the mean response of a thousand others is like trying to forecast the weather on a given date by averaging historic records of that day's weather.

The rise of ‘precision medicine’ signals that customizing treatments is a tough task indeed [24]; instead of using a person's genome to create a bespoke treatment, an oft-cited expectation raised around the time of the first drafts of the human genome in 2000, today, a more practical endeavour is underway to sequence millions of genomes to link genes with pathologies in stratified populations.

Yet rather than controlling us, our genes dance to the tune of higher levels of control, as well as environmental influences (‘downward causation’ in Noble's parlance (see http://musicoflife.co.uk/pdfs/GenesandCausation.pdf)). Thus, both genotypical and phenotypical data are required to reliably formulate aetiological relations. This is now being attempted (see https://www.systemsbiology.org/research/100k-wellness-project/; http://www.humanlongevity.com/science-technology/science/human-genomics/; http://www.genomicsengland.co.uk/about-gecip/about-gecip-data-and-data-access/ but falls foul of the complexity of the feature space and the vastness of the datasets required to achieve statistical significance. Mechanistic theories explaining how living systems work are needed to serve as the architect of the experimental design process itself.

Despite the vast funding now directed at genomics projects, correlations cannot divine the dynamical processes of disease. To understand cancer, for example, one must understand the cell cycle. Despite the 2001 Nobel Prize for identifying key regulators of this cycle, an understanding of the dynamics of cancer remains at an early stage. Although the multistage model of cancer formation is more than half a century old [25], there is still a lack of theory informing daily work in cancer research [26]. There are some examples which demonstrate how modelling gives important insights into clinical applications, such as combination cancer therapies [27]. Nonetheless, such explanatory models will never arise from big data ‘snapshots’ of patient pheno- and genotypes alone, unless a vast number of snapshots are taken over time to chart the temporal changes in the body. Ultimately, structural descriptions—spatio-temporal, mechanistic, pathway models—are necessary, not least to get medicines adopted by regulatory authorities.

## 4. Pre-Galilean rationalization

Our critique of the big data era might lead one to the mistaken view that we believe biology to be devoid of theory. On the contrary, there is considerable mathematical theory pertaining to population dynamics, biophysics, epidemiology, ecology and pathway dynamics, to name a few areas. These pale in comparison with evolutionary biology, where theory abounds.

However, though it is possible to make some predictions [28–31], biological theories have rarely reached the level of generality and power seen in physical theories such as general relativity or quantum chromodynamics. One issue is chaos, as is the case in weather forecasting [32]. Another is that in biological problems the state space is so vast that detailed predictions are going to be elusive. Moreover, mathematics does not have the long-standing relation to the life sciences that it does to the physical sciences, as pointed out by May [33]. Evolutionary theory has been under development since the start of the twentieth century, for instance when a simple equation to show the effect of passing genes down the generations was found by G. H. Hardy and generalized by Wilhelm Weinberg, a relatively short period when compared with physical theories in fields such as mechanics, thermodynamics and gravitation. As a result, the life sciences have few, if any, recognized universal laws (although May went so far as to call the Hardy–Weinberg Law biology's equivalent of Newton's First Law). Even today, there are disputes over key features, such as the evolution of eusociality [34,35].

Because there are evolutionary theories and they often appear reasonable and consistent with our experience, which kind of theories are they? Quite simply, they are a form of pre-Galilean rationalism. As data mining is pre-Baconian in that it is not constrained by experimental design, evolutionary theory is pre-Galilean in that it is not constituted by a mathematical conceptualization of phenomenal behaviour. Yes, we can do (mostly) *post hoc* explanations of evolutionary phenomena but, no, these do not count as science in the modern sense, in which the ultimate test of our knowledge is making predictions that, when tested, are shown to be correct.

## 5. Conclusion

Big data and machine learning are intoxicating to many researchers in biology and medicine because it is hard to develop and use predictive theory in these fields. This is the case even in the relatively simpler disciplines of physics and chemistry. Despite Dirac's aphorism that ‘the underlying physical laws necessary for the mathematical theory of … the whole of chemistry are thus completely known’, we still cannot convincingly work out how the boiling point of liquid water emerges from its molecular properties.

Quantum simulations of material properties performed by different researchers and with different software are now able to produce identical results [36]. They can be used to illuminate disease mechanisms, by use of fundamental chemical theory. As one example, quantum chemical computations can test halocarbons *in silico* to illuminate the origins of liver toxicity [37]. Because different theories apply at different length scales, it is non-trivial to use theory to go from quantum theory to bulk properties. Even so, one of us (P.V.C.) has found a way to solve the multiscale problem to provide predictions of complex materials properties, starting from the parameter-free quantum level, that are borne out by experiment [38,39]. We have also seen examples of model-based predictions of material properties that, for example, have directly influenced industrial processes [40] and also the use of polymers in Blu-ray discs [41].

There are also big data initiatives in chemistry and materials science, following the wider trend across science. Various teams are creating algorithms that learn from past experiments how to make new molecules. The hope is that one day a machine will be built that can synthesize any organic compound. To do this, it would use existing knowledge about how molecules can be built [42], an algorithm that can map out a synthetic route [34,35], and a robotic reactor to carry out the appropriate steps.

One team has devised a machine learning algorithm to predict ways to make crystals of templated vanadium selenites by training the algorithm on data from 4000 attempts to make the crystals under different reaction conditions, ranging from successful experiments to ‘dark reactions’, failed attempts that hold often unreported and thus uninterpreted information (see http://darkreactions.haverford.edu/). Their algorithm could make a crystalline product more reliably than researchers. However, the reasoning that it used to do this is unclear [43].

Like its biological peers, the Materials Genome Initiative (see https://mgi.nist.gov/) is seeking answers by amassing data (the recipe and properties of a material). As discussed, many correlations in all these data are likely to be false leads. That is why a team at Los Alamos is using basic theory to help guide, enhance and refine a variant on the Materials Genome Initiative [44,45]. They direct data collection using Bayesian probabilistic methods, with experimental design driven by theoretical insight.

Given that chemistry and materials science are intermediate in complexity between physics and biology, this approach—a blend of theory and measurement—offers a glimpse of how to make progress in the biological sciences. Along with the examples of weather forecasting and the hunt for the Higgs, it underlines how the biosciences need more mechanistic theories to serve as the architect of the experimental design process [46].

There is now growing recognition that models are important, in precision medicine for example [47]. One of us (E.R.D.) has prioritized experiments for the most efficient design of gene regulatory networks, where the aim is to maximally reduce model uncertainty as it pertains to using the network model for drug design [48]. Another of us (P.V.C.) has reported results that show how to take a person's genetic make-up so as to design an ideal drug to treat them. This approach uses Newtonian dynamics to show how candidate drug molecules interact with a target protein in the body but it is innovative because of its use of Monte Carlo methods [49]. This allows modelling to be more accurate and reproducible because it explores the full range of interactions, because the system is chaotic and probabilistic (a statistical distribution of drug molecule and protein trajectories), and then can be used to work out how likely the drug candidates are to bind. These binding affinities can be calculated using high-performance computing within just a few hours, a development that heralds the development of true personalized medicine.

There are even ambitious efforts to build models of the entire human body. At around atomic resolution, you would need some 10^32^ bits of information to define the body. Much of these data are redundant; however, finding a way to model the body remains a daunting task. Even though great progress has been made, for instance in modelling the heart [50], efforts to create virtual humans [51] will require decades to come to fruition.

Even so, there is a deep-seated cultural problem to overcome. The prevailing view is that the complexities of life do not easily yield to theoretical models. Leading biological and medical journals publish vanishingly little theory-led, let alone purely theoretical, work. As a result of the well-established phenomenon of confirmation bias [52], peer review in journals that specialize in biology and medicine tends to maintain the *status quo*—and that means the current emphasis on pre-Galilean explanations for observations and experiments. Too few biology or bioinformatics students are trained to understand the theory of dynamical systems needed to describe biological processes. Many remain content with *post hoc* rationalizations and are daunted by the prospect of trying to deliver truly actionable predictions in medicine.

More attention needs to be given to theory if the many attempts at integrating computation, big data and experiment are to provide useful knowledge. A substantial portion of funding used to gather and process data should be diverted towards efforts to discern the laws of biology. We look forward to a decline in pre-Baconian thinking and a rise of predictive modelling based on structured experimental design, when students will learn the equations of life as well as Newton's laws and start to model the behaviours of complex biological systems, such as the human body. The field of big data is important but its impact is diminished without big theory.
